# Gradients in low birthweight by maternal education: A comparative perspective

**DOI:** 10.1016/j.ssmph.2024.101674

**Published:** 2024-04-25

**Authors:** Lidia Panico, Alice Goisis, Melissa Martinson

**Affiliations:** a Center for Research on Social Inequalities (CRIS), Sciences Po, CNRS, 27, rue Saint-Guillaume, 75337, Paris, Cedex 07, France; b Institut National d’Etudes Démographiques (INED), 9 cours des Humanités, CS 50004, 93322, Aubervilliers, Cedex, France; c Centre for Longitudinal Studies, Department of Social Science, University College London, 55-59 Gordon Square, London, WC1H 0NU, UK; d School of Social Work, University of Washington, 4101 15th Ave NE, Seattle, WA, 98105-6299, USA

**Keywords:** Health inequalities, Comparative research, Determinants of health inequalities, Birth outcomes

## Abstract

**Background:**

Longstanding research has shown strong inequalities in low birthweight by household income. However, most such research has focused on Anglophone countries, while evidence emerging from other developed countries suggest a stronger role of education rather than incomes in creating inequalities at birth. This paper compares gradients in low birthweight by maternal education, as well as explores underlying mechanisms contributing to these gradients, in France, the United States, and the United Kingdom.

**Methods:**

Analyses are based on harmonized data from large, nationally-representative samples from France, UK and US. We use regression models and decomposition methods to explore the relative role of several possible mechanisms in producing birthweight inequalities.

**Results:**

Inequalities in low birth weight across maternal education groups were relatively similar in the United States, the United Kingdom and France. However, the individual-level mechanisms producing such inequalities varied substantially across the three countries, with income being most important in the US, pregnancy smoking being most evident in France, and the UK occupying an intermediate position. Differences in the mechanisms producing birth health inequalities mirror differences in the policy environment in the three countries.

**Conclusion:**

While inequalities in health appear from the earliest moments in many countries, our results suggest research on birth health inequalities, and therefore policies, is not easily generalizable across national contexts, and call for more scholarship in uncovering the “whys” of health inequalities in a variety of contexts.

## 1 Introduction

The in-utero period is a critical moment of an individual's life, and markers for in-utero processes, such as low birthweight, are strongly correlated with future health and developmental trajectories (Bilgin, Mendonca, & Wolke, 2018; Choi & Martinson, 2018; Ekeus, Lindström, Lindblad, Rasmussen, & Hjern, 2010; Lærum et al., 2017). Socio-economic differences in birth outcomes have remained pervasive across Western countries (Blumenshine, Egerter, Barclay, Cubbin, & Braveman, 2010). For example, research comparing data from the United States (US), United Kingdom (UK), Canada and Australia shows stark gradients in low birthweight (i.e., weight at birth of less than 2500 grams) by household income, with poorer households at higher risk of having a low birthweight infant compared to their richer counterparts (Martinson & Reichman, 2016).

Comparing inequalities across different national contexts allows understanding how such settings moderate the relationship between socio-economic background and health at birth (for an example, see Martinson & Reichman, 2016), and single country studies are difficult to compare, mostly due to differences in study design and methodology. Furthermore, while most comparative research on socio-economic inequalities in birth health has focused on Anglophone countries, evidence shows socio-economic gradients in outcomes such as low birthweight exist in other developed countries, including most European settings (Jansen et al., 2009; Morgen et al., 2017; Panico & Tô, 2023).

In contrast to the Anglophone literature focusing on gradients by household income, many of papers from European settings look at maternal education. It is therefore difficult to compare the relative magnitude, and possible underlying mechanisms, of socio-economic inequalities in birth health across these different settings. This comparison would be substantially and politically interesting both because of the different baseline population inequalities, and because of the different policy environments across these countries and regions.

Therefore, in this paper our first aim is to compare the relative differences in birthweight according to maternal education, using high-quality, harmonized, nationally representative data from three countries with different policy settings and welfare regimes: France (Elfe birth cohort), UK (Millennium Cohort Study, MCS), and the US (Early Childhood Longitudinal Study, ECLS-B). We use parental education as our primary marker of socioeconomic background, rather than household income, based on both theoretical and statistical considerations. Parental education is a good proxy for available household resources and environment, values and beliefs around health, childcare, etc., and the interactions between household members, and has therefore been increasingly used as the main marker of social stratification when child outcomes are concerned (Bradbury, Corak, Waldfogel, & Washbrook, 2015). Furthermore, education is easier to self-report than income, and therefore tends to be measured more precisely. We choose maternal education over paternal education as this measure is particularly relevant for pregnancy processes and birth outcomes, while remaining highly correlated to paternal education.

The second part of the paper focuses on potential underlying mechanisms. While longstanding research compares macro-level processes across countries, a growing literature compares on the role of micro-level mechanisms producing socio-economic inequalities in child health across countries. For inequalities in health at birth, this literature is still growing as the etiological factors driving inequalities in birth health are still not fully understood. First, selection might be at play: more educated women have higher incomes (Tamborini, Kim, & Sakamoto, 2015), are more likely to marry more educated, higher income husbands (Eika, Mogstad, & Zafar, 2019), and are less likely to separate or divorce (McLanahan & Percheski, 2008). Education and other markers of socio-economic advantage may however benefit women beyond increasing incomes and selecting them into more stable, higher income partnerships.

A number of potential mechanisms through which socio-economic disadvantage can influence birth weight have been proposed, mapping the causal pathways through which social variables influence infant health (Mosely and Chen 1984; Gage, Fang, O’Neill, & DiRienzo, 2013). Education enhances critical thinking skills, personal efficacy, and social networking (Kingston, Hubbard, Lapp, Schroeder, & Wilson, 2003; Mirowsky & Ross, 2003), impacting a woman's ability to seek health care, information, and communicate with health care providers, and moderates the effect of poor mental health and stress, which are linked to negative birth outcomes (Kramer, Seguin, Lydon, & Goulet, 2000; Schetter & Tanner, 2012). Indeed, even in countries such as France, with comparatively accessible healthcare services, more disadvantaged women are more at risk of inadequate prenatal care (Gonthier et al., 2017). The experience of stress and of poor maternal mental health is also unequally distributed across socio-economic groups (Meadows, McLanahan, & Brooks-Gunn, 2008). Moreover, education correlates with tobacco and alcohol consumption and other health behaviors during pregnancy (Currie & Moretti, 2003); smoking in particular appears to be particularly important mechanism to understand the production of socio-economic inequalities in health at birth (Panico & Tô, 2023).

Our second aim is therefore to compare the relative importance of a set of micro-level mechanisms, such as pregnancy smoking, household income, antenatal care access, etc., in explaining gaps in birthweight across maternal education groups, in France, UK and US. A novelty of our approach is that we test household income as a potential mechanism rather than the main social stratifier, as much of the previous research has done. This allows treating education as a structural form of parental advantage, which is upstream and is a strong driver of subsequent socioeconomic processes such as income and employment. Our approach therefore tests income as a mechanism through which advantage is transmitted.

Throughout the paper, the international comparison we propose adds a comparative angle to our research questions, and therefore explores the role of the national setting in these relationships. In relation to our first question, the UK and the US have been previously compared in terms of child outcomes (Bradbury et al., 2015; Jackson, Kiernan, & McLanahan, 2017), although rarely for birth outcomes specifically (Martinson & Reichman, 2016). The US and UK provide similar demographic and cultural settings but key differences in the provision of services to families and children, with generally more in generous access to healthcare and welfare in the UK than US (Meyers & Gornick, 2005). France is an important addition to this comparison. While having demographically similar populations, its welfare systems is considered even more generous than the UK, and, unlike the US and the UK's targeted approach, the French system takes a more universal approach, particularly concerning family and child benefits and services (Thévenon, 2011). We hypothesize that countries with more generous child and family services (in our case, France, and to a lesser extent, the UK) should have a weaker relationship between maternal education and birth outcomes, as these countries propose services and benefits that may compensate for a lack of household resources among more disadvantaged families (Esping-Andersen, 2002).

In relation to our second question, there is to our knowledge no work systematically comparing, across different national settings, the relative role of different micro-level mechanisms in producing health inequalities at birth in Western countries. As highlighted before, the national contexts studied differ in important manners, notably the availability, generosity, and targeting of benefits and services. We therefore hypothesize that the relative role of different mechanisms underlying the relationship between maternal education and health at birth will differ across countries, with a more important role of financial household resources in the US given the lower availability and level of state services and benefits compared to France and the UK.

## 2 Data and methods

### 2.1 Data and sample selection

Analyses rely on three large, nationally representative studies with comparable, rich data on family characteristics, birth outcomes, and pathway variables. Their key characteristics are described below.

The Etude Longitudinale Française depuis l’Enfance (**Elfe**), France's first representative, large-scale multidisciplinary birth cohort, follows about 18,000 children born in 2011 at a representative sample of 344 hospitals in continental France (Charles et al., 2020). Data collected shortly after birth in hospital (including data from the mothers' pregnancy notes) and two months after birth are used for analyses. The Millennium Cohort Study (**MCS**) is a cohort of over 19,000 children born in 2000–2 and living in the UK shortly after birth (Connelly & Platt, 2014). We use data from interviews with the carers when the child was around 9 months (including retrospective information about the pregnancy and birth, and hospital registration records). The Early Childhood Longitudinal Study-Birth cohort (**ECLS-B**) follows a nationally representative sample of 10,700 children born in 2001 (Snow et al., 2007). Data used here were collected with the main carer through interviews when the child was about 9 months. Information on health outcomes at birth was taken from the birth certificate.

Elfe does not include births at less than 33 weeks’ gestation. To ensure comparability across the studies, we therefore exclude from all our analyses births at less than 33 weeks gestation and triplets. Because of the ECLS-B oversampling strategy (most relevant here, ECLS-B oversampled low and very low birthweight infants, as well as twins), this exclusion criteria does involve dropping a substantial number of cases from this sample. These restrictions imply that results presented here for the ECLS-B, and to a smaller extent, for the MCS, might slightly under-estimate the real educational gradients in birthweight.^1^ We therefore run sensitivity analyses for MCS and ECLS-B including births at 33 weeks gestation and lower (see Appendix, Table 1A and 2A) to check the robustness of our results.

Elfe only includes mothers aged 18 and over for consent issues. We chose not to apply this restriction to ECLS-B and MCS. In France, births under 18 are extremely rare, therefore the exclusion does not substantively change the potential French sample: according to data from the national civil registry, in 2011, only 0.5% of births in continental France were to mothers aged under 18 at birth (Insee, 2017). For the UK and the US, this restriction would bias our estimations, particularly those relating to the less advantaged education groups. In 2000, when the cohort members were born, 2.3% of all births in the UK were to mothers under the age of 18 (ONS, 2018), and 3% in the USA (Centre for Disease Control, 2002).

We carry out complete case analyses, producing unweighted analytical samples of 12,238 cohort members for Elfe, 15,871 for MCS and 8550 for ECLS-B. In the sensitivity analyses, we check if our baseline model changes substantially when using non-complete (see Appendix, Table 1A).

### 2.2 Measurements

Our **key outcome variable** is low birthweight, modelled as a binary variable, indicating whether the cohort child weighted 2500 g or less at birth. Furthermore, in initial descriptive analyses, we also consider mean birthweight (in grams), mean gestational age (in days), and prematurity (a binary indicator capturing whether the cohort child was at less than 37 weeks gestation), to fully describe our analytical samples.

We run a number of specification checks to ensure our results are robust to different specifications of birthweight. First, we run models using birthweight as a continuous measure, using linear regression models (see Appendix, Table 1A). Second, because of the negative outcomes linked to high birthweights, we exclude births at or over 4,5 kilos from the reference (“normal” weight) category when using the binary indicator for low birthweight (see Appendix, Table 1A). This excludes about 1% of the ECLS-B analytical sample, about 1.7% for MCS, and about 1% for Elfe.

Our **main independent variable** is education. We choose parental education over other markers of socio-economic status (SES) such income or occupational attainment, for three main reasons. First, parental education is a relatively stable measure over a parent's lifecourse, and this is particularly important for outcomes around birth, when the incomes and careers of parents are in flux. Second, education allows focusing on a human capital perspective, where education is viewed as an investment that can yield a range of returns, for example, on the labour market, but also in terms of being able to access health care and information on health behaviours. From this perspective, parental education can be seen as a structural form of parental (dis)advantage, and while other factors, such as income and smoking, can be conceptualised as mechanisms through which parental advantage can be transmitted (Olczyk et al., 2021). Finally, parental education is less subject to measurement error than income, is more easily harmonizable, and is substantively more salient for a cross-country comparison than other SES indicators. As a result, it has been used by several projects comparing child health and development across countries (Bradbury et al., 2015; Panico et al., 2023).

We choose maternal education over paternal education as this measure is particularly relevant for pregnancy processes and birth outcomes. For example, work by Swaminathan et al. (2022) found that maternal education was associated with birth health, and that this association was more robust than paternal education. Furthermore, maternal education is strongly correlated with paternal education (De Hauw, Grow, & Van Bavel, 2017; Schwartz, 2013; Schwartz & Mare, 2005), and, in all three studies, maternal education is much less likely to be missing than father's education. In fact, in the three studies, father's education is not missing at random: more disadvantaged households and single parent households are less likely to report information on father's education.

Based on other comparative work on socio-economic inequalities in early child outcomes (Bradbury et al., 2015; Panico et al., 2023), in our main analyses we distinguish three broad maternal education groups: high, equivalent to a bachelor's degree or more; medium, equivalent to some college or post-secondary vocational qualifications; and low, equivalent to a high school diploma or less. In the UK, we classify A-levels in the medium category: while A-level study normally takes place between ages 16 and 18, when high school diplomas are usually prepared, authors comparing the British and US system (Brabury et al., 2015) have argued that A-levels have more in common with a first year of a US college degree than high school. This is because students can only access A-level study if they have attained adequate grades at GCSE (typically 5 or more GCSEs at grade C or above, achieved by only about half the population). Furthermore, A-level study is specialized around 3 to 4 academic subjects, and therefore covers relatively advanced material. In sensitivity analyses, in France and the US, we also reclassify high school diplomas and equivalent in the medium education group (the low education group therefore containing only qualifications lower than a high school diploma; see Appendix, Table 1A).

We explore a number of potential micro-level **mechanisms** to explain birthweight gradients by maternal education; our choice of mechanisms to test in our models is guided by the existing literature on socio-economic inequalities in birthweight, as reported above. First, we look at the role of household income, modelled as quintiles of equivalised household income (income equivalisation using the OECD-modified scale). Second, we explore maternal smoking during the third trimester of the pregnancy, treated as a binary dummy variable (1 = any smoking during the third trimester) in descriptive analyses and a continuous variable measuring the number of cigarettes per day in regression analyses (no smoking is coded as 0 cigarettes). We use third trimester smoking because of data availability in the ECLS; this probably means that we are only capturing the most persistent smokers who did not quit earlier in the pregnancy. Alcohol consumption during the pregnancy is measured as a dummy for any alcohol consumption during the pregnancy (1 = yes). To capture maternal health before the pregnancy, we include her pre-pregnancy BMI, categorised using WHO (2000) guidelines of underweight, normal, overweight and obese. These variables are self-reported in all surveys. Any pregnancy complications during pregnancy is a dummy variable taking the value of 1 if any complications were reported from the following: gestational diabetes, hypertension, eclampsia and pre-eclampsia, placenta previa. For MCS, the only available information is whether the mother had any illnesses or problems during the pregnancy. This information is self-reported for MCS; extracted from the mother's medical notes in Elfe; and from the birth certificates in ECLS-B. These factors are known to be underreported on birth certificates (i.e., for ECLS, these variables are likely to have high specificity but low sensitivity); this is less likely to be the case for data extracted directly from the mother's medical notes. It is therefore likely that we underestimate pregnancy complications in the ECLS data. Whether antenatal care was started from the first trimester (1 = no) was only available for MCS and ECLS-B and therefore was only added to separate models (see Appendix, Table 1A).

Finally, we include a number of covariates as **controls**, including: Child sex (1 = male); child parity (1 = first-born); multiple pregnancy indicator (1 = twin/triplets); mother's height (continuous); whether mother worked during pregnancy (1 = yes); marital status of parents at birth (1 = unmarried at birth); maternal age at birth (continuous) and a squared term of maternal age. Because maternal age at birth and marital status could be too closely tied to maternal education, in separate models we exclude these variables to check that they do not overly impact our coefficients of interest (see Appendix, Table 1A). In separate models for Elfe and ECLS-B, we also control for maternal nativity (1 = foreign-born); this variable was not harmonizable for the MCS (see Appendix, Table 1A).

### 2.3 Statistical analyses

We model low birthweight using a series of logistic regression models and report the resulting Odds Ratios. Kuha and Mills (2018) argue that, in most circumstances, it is not problematic to compare ORs across model specifications, and indeed such frameworks allow for more easily interpretable results. We run five models: Model 0 (unadjusted model) regresses low birthweight on maternal education*.* Model 1 includes the basic child and household covariates (child sex, child parity, multiple birth, maternal height, maternal work, maternal age at birth, and marital status); Model 2 adds household income; Model 3 includes maternal health behaviours and health status (alcohol during the pregnancy; smoking during the pregnancy; maternal BMI; pregnancy complications); and finally Model 4 includes all covariates.

In a second step, to explore the mechanisms and compare the relative importance of the different mechanism variables, Kitagawa-Oaxaca-Blinder (KOB) two-way decomposition analyses are carried out (Blinder, 1973; Kitagawa, 1955; Oaxaca, 1973), decomposing the low birthweight gap between the low and high education groups, the medium and low education groups, and finally the medium versus high education groups.

To check that our results are not driven by specific population groups, we carry out a number of sensitivity analyses by exploring whether the associations are different across ethnic/racial groups, for the ECLS-B sample we restrict to the US non-Hispanic white population; for UK, we restrict to White British; for France, where data on ethnicity/race cannot be collected, we restrict to mothers born in France with French nationality from birth (see Appendix, Table 1A).

Like many surveys, selection into our studies is not random and more advantaged families are more likely to participate. Survey weights are used in all analyses to correct for both non-random sampling design, and for non-response to the survey. Weights are derived by the study teams (Bethel, Green, Kalton, & Nord, 2005; Plewis, 2007; Simeon, 2019). STATA statistical software was used to conduct all analyses.

## 3 Results

The first panel of Table 1 shows that the overall proportion of low birthweight in our analytical samples is higher in the US, and lowest in France. Interestingly, while the mean birthweight and mean gestational age are relatively similar across the three countries, the proportion of low birthweight and prematurity is not, suggesting distributional differences in birth outcomes across the three countries, i.e., the distribution of birthweight and especially of gestational age is more skewed to the left and flatter in the US compared to France.

The two subsequent panels of Table 1 report the child and household characteristics that we control for in our models. Our analytical samples are relatively similar in terms of child characteristics across the three surveys, except for a higher proportion of twin births in the US and France (2.7 and 2.3% respectively) than the UK (1.2%). As expected, the table shows a larger proportion of highly educated mothers in France, compatible with a massification of tertiary education in France over the last decades, and a lower proportion of teenage mothers, as discussed above. Differences across countries are also evident on some of the mechanism variables (Table 1, fourth panel), in particular for alcohol consumption during the pregnancy (most reported in the UK and least in the US), and pregnancy complications (again, most reported in the UK and least in the US).^2^

Table 2 reports the Odds Ratios from logistic regression models predicting low birthweight in each country by comparing mothers with low education and high education to those with medium education (reference category). The first two models (Model 0, with no basic controls, and Model 1, with basic controls) show that gradients in low birthweight by maternal education are evident in the three countries, although slightly larger in magnitude in the UK than in France and the US. In the US and France, low education groups have a 25% higher risk of low birthweight compared to the mid-education group, while in the UK they have a 50% higher risk. The magnitude of the low birthweight gap between middle and high education groups is largest in the UK (OR = 0.65) and smallest in the US (OR = 0.88), with France in an intermediate position (OR = 0.74).

Going from Model 0 to Model 1 (i.e. when basic child and household covariates are added to the unadjusted model) did not change substantively the results for the UK and US, but did increase the magnitude in the relative gap in France, so that relative differences in birthweight are now similar to those found in the UK. This is driven by the low versus middle education groups comparison. Odds Ratios for this comparison in France go from 1.21 in Model 0 to 1.51 to Model 1, similar to the UK's OR of 1.44. Differences in birthweight across maternal education groups are statistically significant in all countries, except for the difference between high and medium education groups in the US in Model 0 (although it becomes significant in Model 1).

Introducing income (Model 2) to the model reduces the Odds Ratios most in the US (although this reduction is only evident for the low versus medium education term) and least in France and the UK, while the inclusion of health behaviour and health status variables (Model 3) reduce coefficients most in France and the UK (although this reduction is only evident for the low versus medium education terms) and least in the US. In fully adjusted models (Model 4), educational differences in low birthweight are no longer statistically significant in the US, suggesting the model is capturing most of the likely underlying mechanisms. Differences in France and the UK are reduced but remain statistically significant for the low versus medium education comparison, and not modified for the medium versus high education comparison, suggesting that for these countries and particularly for the medium vs high education group, unobserved mechanisms are likely to play a role in explaining low birthweight differences across maternal education groups.

In a second part to our analyses, we further delve in the relative role of different micro-level mechanisms in producing birth health inequalities by using decomposition techniques (Table 3). Results from the US are relatively simple to interpret: the gap in birthweight between the three education groups are entirely driven by compositional differences between the groups (the “explained” term), and this term is in turn almost entirely driven by compositional differences in equivalised household income. In the comparison between low versus medium, and medium versus high education groups, distributional differences in tobacco consumption during the pregnancy also play a small role.

In the UK, both distributional differences in the covariates (the “explained” term) and heterogeneous effects of covariates on the outcomes (the “unexplained” term, i.e., whether the association between a covariate, such as income, on low birthweight is different across education groups, and that difference accounts for the overall gap in low birthweight between education groups) matter. Unlike the US, many mechanisms emerge for the UK: for example, for the low birthweight gap in low versus high education group, and for the low versus medium comparison, tobacco consumption during the pregnancy is the most important mechanism, followed by income. However, for medium versus high education comparison, no mechanism comes across as particularly important.

Finally, in France, depending on the comparison, sometimes the “explained” term is the only one that is significant (low versus medium education comparison), sometimes the “unexplained” (medium versus high education), and sometimes both (low versus high education). The distributional difference in tobacco consumption was the most important mechanism across all comparisons, although occasionally differences in pregnancy complications also emerged. To further explore this finding, Table 2A in the Appendix reports smoking rates by maternal education for the three countries, as well as average number of cigarettes smoked conditional on any smoking. These results show that the educational gradients in smoking are similar in the three countries, with more educated mothers smoking less than less educated mothers. However, among mothers who smoke, the educational patterns in the number of cigarettes smoked was different across the three countries: there was no educational gradient in the US; in the UK, there was only a statistically significant difference between the low education group (who smoked on average more than two other more educated groups) and the rest; the social gradient was most evident in France: conditional on any tobacco consumption during the pregnancy, more educated mothers smoked significantly less than less educated mothers, and differences were significant across all education groups.

We carry out several specification checks and supplementary analyses to test the robustness of our results. All supplementary analyses can be found in Table 1A in the Appendix. First, as maternal age and marital status might be collinear with maternal education, in supplementary models, we exclude these variables, which slightly accentuate inequalities in low birthweight for all three studies but particularly the US, suggesting our main estimates are a conservative representation of real gaps. To further address concerns that maternal age, marital status, and education are highly correlated, we estimate supplementary models including only mothers aged over 25 years of age. We don't observe substantive change in the magnitude of the primary results for education differences in low birthweight, although, as we reduce the sample by as much as a third (as is the case for the US sample), there is an impact on the statistical significance of our estimates.

Furthermore, some salient variables could not be harmonized across all countries: Maternal nativity was only available in the US and France. Including this variable increased education gaps in the US, but not in France. Adding antenatal care to Models 3, for the US and UK samples only, did not change substantially estimates for either country.

Second, we check that our results are robust to our sample selection. First, we run Model 0 and Model 1 on samples including all cases with information on education, birthweight, and basic controls (i.e., we include cases that might have missing information on income, health behaviours or health status). Results are very similar to our complete case analysis for the US; ORs are reduced for the UK for the low vs medium education comparison (no change for the medium vs high comparison); and slightly increased for France. Including, in the UK and US samples, very premature children (children born below 33 weeks gestations are not surveyed in the French study) does not change results for the US; for the UK, they slightly reduce the ORs for the low vs medium education comparison, and slightly increase them for the medium vs high comparison. Finally, for the US and UK we run analyses excluding mothers aged 18 and under (in order to match to the French sample, where mothers under the age of 18 were not surveyed). The estimates from this analysis are virtually the same as for our main models, with however some loss of statistical significance in the US for the low versus medium education comparison, probably because the sample size drop is particularly relevant for the low education group.

Third, we check that our results are robust to different specifications of our birthweight outcome. First, we use linear regression models and a continuous measure of birthweight. Results again suggest a similar magnitude in the overall gradient in mean birthweight by maternal education across the three countries, but with more similar gaps in the US and the UK and slightly smaller gaps in France. In the full model of this specification, in France and the UK, the coefficients remain slightly statistically significant. We also use our main specification (logistic regression models predicting odds ratios of low birthweight) but exclude births at 4500 g or heavier from the reference category. This does not modify substantively results in any country.

Finally, we re-run analyses in a sample excluding minority racial and ethnic groups, to check that country differences are not due to heterogeneous distributions of population subgroups or within-country racial and ethnic birth outcomes differences. Results are robust to this check.

## 4 Discussion

Using comparable, nationally-representative studies, this paper examines how and why different patterns in the relationship between socio-economic status and low birthweight exist in France, the UK, and the US. A first focus of this paper was to explore the strength of the relationship between maternal education and low birthweight across these three national settings. Differently from the comparative literature on socio-economic inequalities in birth and child health outcomes, which tends to use income to measure socio-economic disadvantage (Avendano & Kawachi, 2011; Martinson & Reichman, 2016; Martinson, Teitler, & Reichman, 2011), we focus on maternal education as a structural marker of socioeconomic (dis)advantage. We find that all three countries have evident socio-economic inequalities in low birthweight, and that this is robust to various ways of modelling education and birth weight.

Contrary to our initial hypothesis, we do not find that the US has starker gaps in low birthweight between the most and least educated compared to France and the UK. However, this finding needs to be considered in light of two facts: first, the US does appear to have the worst overall performance in terms of birth outcomes. Second, because we compare different cohorts of parents (youngest in France, oldest in US and UK), and because of very different education policies in the three countries, our disadvantaged group (the “low” education group) is largest in the UK, closely followed by the US, and smallest in France, where the massification of tertiary education for more recent cohorts means that over half of mothers in our sample hold tertiary qualifications. Therefore, while the relative magnitude of the gaps in low birthweight by education groups is similar in the three countries, the size of the population at risk of poor birth outcomes is larger in the US and UK than in France, and is most meaningful in the US, given its high overall proportion of low birthweight births.

This result calls for two reflections: first, our results suggest that different markers of socio-economic background may produce different patterns of health inequalities, and therefore researchers should be careful with their conceptualisation of “socio-economic” disadvantage, especially when comparing across countries. Theoretical and empirical considerations raise concerns about how socio-economic status is measured and interpreted in health inequalities research. Socio-economic inequalities in health define groups in a population who are disadvantaged to such an extent that it affects their opportunity to achieve good health (Bartley, 2016). This disadvantage can be defined in a number of ways, for example, by a person's financial resources, their social position, their gender or ethnicity. Different ways of defining socio-economic status can give rise to different relationships with health outcomes, including depending on the (national) context. These reflections might be particularly salient for comparative research: our results suggest that using income versus education as the main stratifier might create different cross-country patterns of birth health inequalities.

Second, our results should be couched within their historical and lifecourse settings: given our birth cohorts (for the US and UK, children born at the turn of the millennium; for France, children born in 2011) and that our outcomes reflect in-utero processes, it is important to point that in spite of significant differences in social policies across these three countries, the mothers of the cohort members would have experienced relatively similar access to health care during the pregnancy: the expansion of Medicaid (a public health insurance program for households on low income) in the US in late 1980s meant that nearly 96% of births in the US were covered either by Medicaid or private insurance by the late 1990s, with coverage concerning both the antenatal period and infancy (Provost & Hughes, 2000). In the UK, the National Health Service (NHS) provides additional access to all pregnant women, including free prescriptions and dental care; the NHS also provides relevant preconception care to most women. In France, social security and both private and public insurance programs mean that most pregnant women have access to comprehensive and affordable care during the preconception period, pregnancy and at birth. Therefore, while maternal and infant mortality remains historically higher in the US than other OECD countries, including the UK and France (OECD, 2022), pregnancy is arguably the lifecourse stage with most comparable access to healthcare across the three countries and may explain the relatively muted differences we find in low birthweight *inequalities* between the three countries.

Our second main aim was testing the relative roles of key micro-level mechanisms in producing birthweight inequalities, and whether these mechanisms differ across our three national settings. Because of our focus on education as the main socioeconomic stratifier, we can explore income as a mechanism to reproduce disadvantage. This is a novelty on much of the literature, which tends to use income as the main socioeconomic indicator. Yet, from a lifecourse perspective, processes shaping household income tend to happen downstream of education. Unlike our first aim, here we do find clear national differences, which can be interpreted in light of different social and health policy contexts across the three countries.

The starkest difference in mechanisms across the three countries concerned income: as we hypothesised, equivalised household income was a clear mechanism in producing low birthweight inequalities across education groups in the US, while it was not an important mechanism in the UK and France. The US has significantly higher child poverty rates than France and the UK (OECD, 2021), two countries that are (or was in early 2000s, in the case of the UK) characterised by relatively generous, near-universal, public cash transfers, particularly during pregnancy and early childhood, as well as other financial mechanisms such as unemployment benefit. For the UK, many such transfer mechanisms, such as Child Benefit and Working Families’ Tax Credit, phased in 1999 with the aim to tackle child poverty and decrease in-work poverty, would have been relevant for households in our cohort, but have been since phased out or significantly curtailed since (Brewer & Hoynes, 2019). Given these reflections, the (temporary) introduction in 2022 of a child allowance in the US (Shaefer et al., 2018) may be particularly salient for child health inequalities in the US.

The differential importance of smoking (which, in our results, emerges most clearly in France compared to the UK and US) could be due to two factors. First, while the educational gradients in any smoking during the pregnancy were similar in the three studies, the educational gradient on the number of cigarettes smoked was starkest for France. These descriptive results suggest that the relative importance of smoking in France might be driven by the intensive rather than extensive margins in smoking (i.e., how much mothers smoke, rather than how many mothers smoke). Cross country differences might reflect different social norms and attitudes towards smoking, as well as public health priorities. France has one of the highest smoking prevalence rates in the Western world, including during pregnancy, despite some of the highest cigarette prices in the world (in contrast, the US has some of lowest rates, despite some of the lowest prices, with the UK occupying a middle position both in terms of smoking rates and prices; Cutler & Glaeser, 2006). France's smoking rates stagnated until relatively recently, in contrast to early declines since the 1960s in the US, and while laws had been brought in throughout the 1990s and 2000s (Pasquereau et al., 2018), an effective and comprehensive tobacco control policy (including plain tobacco packaging, graphic health warnings on tobacco products, smoking cessation programs, and an increase in tobacco pricing) was only introduced in 2016 (after the birth of our cohort members). Indeed, French respondents are less likely to report that they believe smoking is very harmful, followed by the UK, while the US has the highest rates of respondents believing in the harm of tobacco smoking (Cutler & Glaeser, 2006). A change in these attitudes appears to be only very recent in France, after the introduction of the 2016 measures (El-Khoury, Bolze, Gomajee, White, & Melchior, 2019).

Our study provides a unique comparison of the socio-economic inequalities in health at birth across three countries. Our results should however be interpreted in the light of several limitations. First, while low birthweight is a widely used indicator of infant health, particularly for comparative research, and we do provide a number of supplementary analyses specifying our outcome in different manners, we recognize that health at birth can be measured in more nuanced ways (for example, current research explores measures such as foetal head, abdominal and femur size and growth, Conti et al., 2020), although such measurements are often difficult to capture in large, population-based surveys. Second, for France we are not able to include children born under 33 weeks gestation. However, supplementary analyses for the UK and US show that their inclusion does not substantively change our main results, and separate work has shown that very premature births in France do not appear to differ from other births in terms of maternal education, single parenthood, or occupational class (although they are more likely to be born to mothers not of French nationality, aged over 40, and unemployed; Germany et al., 2015). Third, selection into our three surveys is not random, with more advantaged families more likely to participate, a common issue across surveys (Bonevski et al., 2014). Furthermore, because of the sample exclusions we apply to allow comparability between studies (limiting to births over 33 weeks gestation in the US and UK), it is likely that we underestimate the overall low birthweight rates, and possibly the magnitude of the low birthweight gap between more advantaged and more disadvantaged groups and the role of some of the mechanisms in producing these inequalities. Because the focus of this paper is a *comparison* between countries, these issues become problematic for our substantive conclusions only if selection into the studies and into our analytical samples differs across the three countries. This does not look the case. For example, the magnitude of the underestimation of low birthweight in MCS (6.9% in 2010 according to administrative data and 5% in our sample) is similar to Elfe (6.4% in 2010 according to administrative data, and 4.1% in our sample; Euro-Peristat Project, 2013). Fourth, due to difficulties in harmonizing variables across three studies, we are not able to explore the role of several potential mechanisms, such as maternal stress, mental health and social support during the pregnancy. Finally, because of sample sizes and comparability issues, we can only provide results for the overall population, and, in supplementary analyses, for majority groups, but not for minority groups.

## 5 Conclusion

Inequalities in low birth weight across maternal education groups were relatively similar in the United States, the United Kingdom and France. However, the individual-level mechanisms that produce such inequalities varied substantially across countries, with income being most important in the US, and pregnancy smoking being most evident for France. Differences in the mechanisms producing birth health inequalities mirror differences in the policy environment and priorities in these three countries. Our results therefore suggest that public health policies are not easily generalizable across national contexts, and should be tailored to the specificities of each context. Our work calls for more context-specific scholarship in uncovering the “whys” of health inequalities across a larger variety of settings.

## Funding

This work was partly supported by the 10.13039/501100001665Agence Nationale de la Recherche (ANR) [grant number ANR-19-CE41-0003]; the Transatlantic Research Partnership, a program of 10.13039/100009879FACE Foundation and the French Embassy [grant number TJF-2020-62]; 10.13039/100007472UK 10.13039/501100000269Economic and Social Research Council [grant number ES/M001660/1]; a 10.13039/100009633Eunice Kennedy Shriver National Institute of Child Health and Human Development research infrastructure grant [grant number P2C HD042828] to the Center for Studies in Demography & Ecology at the 10.13039/100007812University of Washington. The content is solely the responsibility of the authors and does not necessarily represent the official views of the National Institutes of Health.

## CRediT authorship contribution statement

**Lidia Panico:** Writing – review & editing, Writing – original draft, Project administration, Methodology, Investigation, Funding acquisition, Formal analysis, Data curation, Conceptualization. **Alice Goisis:** Writing – review & editing, Methodology, Investigation, Formal analysis, Conceptualization. **Melissa Martinson:** Writing – review & editing, Methodology, Investigation, Funding acquisition, Formal analysis, Data curation, Conceptualization.

## Declaration of competing interest

The authors declare that they have no known competing financial interests or personal relationships that could have appeared to influence the work reported in this paper.

## Data Availability

The authors do not have permission to share data.

## Footnotes

1 We exclude 11.5% of the sample for the ECLS-B. The excluded cases have a slight pattern by maternal education (excluded births are slightly more likely to be to less educated mothers); however, differences by maternal education were only marginally significant (p = 0.067) between the highest and the lowest educational category; differences with the medium education category were not significant. For MCS, this exclusion concerns 2.8% of the sample. Differences by maternal education were slightly more significant for MCS than ECLS-B (p = 0.0012).

2 As noted in the previous section, the pregnancy complication question is different in the UK compared to the variables available for the French and US data.
