# Supplementary material for: Gradients in low birthweight by maternal education: A comparative perspective
Source: SSM Popul Health. 2024 Apr 25;26:101674. doi: 10.1016/j.ssmph.2024.101674 (PMC11070621; doi:10.1016/j.ssmph.2024.101674)
Supplement: Multimedia component 1 [file mmc1.docx]

| **Socio-economic gradients in low birth weight: a comparative perspective**  **Appendix Table 1A – Sensitivity analyses** | | | | | | | |  |  |  |  |  |
| --- | --- | --- | --- | --- | --- | --- | --- | --- | --- | --- | --- | --- |
|  |  | USA - ECLS | | UK - MCS | | France - Elfe | |  |  |  |  |  |
| *Panel A: Logistic regression, Odds Ratios of low birthweight, overall population* | | | | | | | |  |  |  |  |  |
|  |  | Including births less than 33 weeks | | | | | |  |  |  |  |  |
|  |  | Model 0 | Model 4 | Model 0 | Model 4 | Model 0 | Model 4 |  |  |  |  |  |
| Low education | | 1.20*** | 1.09 | 1.39*** | 1.20** | n/a | n/a |  |  |  |  |  |
| High education | | 0.86* | 0.86 | 0.71** | 0.74** | n/a | n/a |  |  |  |  |  |
|  |  |  |  |  |  |  |  |  |  |  |  |  |
| N |  | 9850 | 9850 | 16270 | 16270 | n/a | n/a |  |  |  |  |  |
|  |  | Excluding mothers aged under 18 at birth | | | | | |  |  |  |  |  |
|  |  | Model 0 | Model 4 | Model 0 | Model 4 | Model 0 | Model 4 |  |  |  |  |  |
| Low education |  | 1.23** | 1.16 | 1.51*** | 1.44** | n/a | n/a |  |  |  |  |  |
| High education |  | 0,88 | 0.83 | 0.65** | 0.67** | n/a | n/a |  |  |  |  |  |
|  |  |  |  |  |  |  |  |  |  |  |  |  |
| N |  | 8300 | 8300 | 15489 | 15489 | n/a | n/a |  |  |  |  |  |
|  |  | Excluding mothers aged under 25 at birth | | | | | |  |  |  |  |  |
|  |  | Model 0 | Model 4 | Model 0 | Model 4 | Model 0 | Model 4 |  |  |  |  |  |
| Low education |  | 1.25* | 1.19 | 1.59*** | 1.52** | 1.26* | 1.36** |  |  |  |  |  |
| High education |  | 0.87 | 0.81 | 0.69** | 0.70** | 0.71*** | 0.75** |  |  |  |  |  |
|  |  |  |  |  |  |  |  |  |  |  |  |  |
| N |  | 5500 | 5500 | 11660 | 11660 | 11192 | 11192 |  |  |  |  |  |
|  |  | Non-complete case model | | | | | |  |  |  |  |  |
|  |  | Model 0 | Model 1 | Model 0 | Model 1 | Model 0 | Model 1 |  |  |  |  |  |
| Low education | | 1.24*** | 1.25** | 1.32*** | 1.29** | 1.34*** | 1.61*** |  |  |  |  |  |
| High education | | 0.88 | 0.75** | 0.66** | 0.67** | 0.79** | 0.81** |  |  |  |  |  |
|  |  |  |  |  |  |  |  |  |  |  |  |  |
| N |  | 8950 | 8950 | 17938 | 17938 | 15182 | 15182 |  |  |  |  |  |
|  |  | Excluding high birthweight (>=4500 grs) from reference category | | | | | |  |  |  |  |  |
|  |  | Model 0 | Model 4 | Model 0 | Model 4 | Model 0 | Model 4 |  |  |  |  |  |
| Low education | | 1.25*** | 1.15 | 1.50*** | 1.29** | 1.26** | 1.35** |  |  |  |  |  |
| High education | | 0.87 | 0.83 | 0.65** | 0.65** | 0.74** | 0.81** |  |  |  |  |  |
|  |  |  |  |  |  |  |  |  |  |  |  |  |
| N |  | 8500 | 8500 | 15 600 | 15 600 | 12126 | 12126 |  |  |  |  |  |
|  |  | Alternative education categories (high school diploma in middle ed. category) | | | | | |  |  |  |  |  |
|  |  | Model 0 | Model 4 | Model 0 | Model 4 | Model 0 | Model 4 |  |  |  |  |  |
| Low education | | 1.08 | 0.95 | n/a | n/a | 1.61*** | 1.45** |  |  |  |  |  |
| High education | | 0.77*** | 0.79** | n/a | n/a | 0.69*** | 0.71*** |  |  |  |  |  |
|  |  |  |  |  |  |  |  |  |  |  |  |  |
| N |  | 8550 | 8550 | n/a | n/a | 12238 | 12238 |  |  |  |  |  |
|  |  | Including prompt antenatal care to M3 & M4 | | | | | |  |  |  |  |  |
|  |  | Model 3 | Model 4 | Model 3 | Model 4 | Model 0 | Model 4 |  |  |  |  |  |
| Low education | | 1.22** | 1.14 | 1.33** | 1.29** | n/a | n/a |  |  |  |  |  |
| High education | | 0.75** | 0.84 | 0.65*** | 0.65*** | n/a | n/a |  |  |  |  |  |
|  |  |  |  |  |  |  |  |  |  |  |  |  |
| N |  | 8550 | 8550 | 15 871 | 15 871 | n/a | n/a |  |  |  |  |  |
|  |  | Excluding maternal age and marital status from controls | | | | | |  |  |  |  |  |
|  |  | Model 1 | Model 4 | Model 1 | Model 4 | Model 1 | Model 4 |  |  |  |  |  |
| Low education | | 1.37*** | 1.18* | 1.46*** | 1.26** | 1.53*** | 1.38*** |  |  |  |  |  |
| High education | | 0.76*** | 0.87 | 0.67** | 0.69** | 0.76*** | 0.81** |  |  |  |  |  |
|  |  |  |  |  |  |  |  |  |  |  |  |  |
| N |  | 8550 | 8550 | 15 871 | 15 871 | 12238 | 12238 |  |  |  |  |  |
|  |  | Including mother nativity to controls | | | | | |  |  |  |  |  |
|  |  | Model 1 | Model 4 | Model 1 | Model 4 | Model 1 | Model 4 |  |  |  |  |  |
| Low education | | 1.38*** | 1.18* | n/a | n/a | 1.50*** | 1.35*** |  |  |  |  |  |
| High education | | 0.76** | 0.87 | n/a | n/a | 0.76*** | 0.81** |  |  |  |  |  |
|  |  |  |  |  |  |  |  |  |  |  |  |  |
| N |  | 8550 | 8550 | n/a | n/a | 12238 | 12238 |  |  |  |  |  |
|  |  |  |  |  |  |  |  |  |  |  |  |  |
| *Panel B: Linear regression, coefficients predicting continuous birthweight (in grams) or low birthweight (binary), total sample* | | | | | | | |  |  |  |  |  |
|  |  | USA - ECLS | | UK - MCS | | France - Elfe | |  |  |  |  |  |
|  |  | Same as main specification, outcome is continuous birthweight (in grams) | | | | | |  |  |  |  |  |
|  |  | Model 0 | Model 4 | Model 0 | Model 4 | Model 0 | Model 4 |  |  |  |  |  |
| Low education | | -84.9*** | -27.1 | -88.4*** | -37.3** | -18.5** | -18.9* |  |  |  |  |  |
| High education | | 13.7 | -20.1 | 27.5 | 28.4* | 26.4** | 22.3** |  |  |  |  |  |
|  |  |  |  |  |  |  |  |  |  |  |  |  |
| N |  | 8550 | 8550 | 15 871 | 15 871 | 12 238 | 12238 |  |  |  |  |  |
|  |  | Same as main specification, outcome is binary low birthweight | | | | | |  |  |  |  |  |
|  |  | Model 0 | Model 4 | Model 0 | Model 4 | Model 0 | Model 4 |  |  |  |  |  |
| Low education |  | 0.012** | 0.006 | 0.020*** | 0.10* | 0.010** | 0.010** |  |  |  |  |  |
| High education |  | -0.006 | -0.008 | -0.14** | -0.14** | -0.010** | -0.006** |  |  |  |  |  |
|  |  |  |  |  |  |  |  |  |  |  |  |  |
| N |  | 8550 | 8550 | 15 871 | 15 871 | 12 238 | 12 238 |  |  |  |  |  |
| *Panel C: Logistic regression, Odds Ratios of low birthweight, subpopulation analyses* | | | | | | | |  |  |  |  |  |
|  |  | USA - ECLS | | UK - MCS | | France - Elfe | |  |  |  |  |  |
|  |  | Non-Hispanic White mothers only | | White mothers only | | French born mothers, of French born parents | |  |  |  |  |  |
|  |  | Model 0 | Model 4 | Model 1 | Model 4 | Model 1 | Model 4 |  |  |  |  |  |
| Low education | | 1.34** | 1.27* | 1.47*** | 1.30** | 1.22*** | 1.17 |  |  |  |  |  |
| High education | | 0.84 | 0.78* | 0.66** | 0.65** | 0.67** | 0.70** |  |  |  |  |  |
|  |  |  |  |  |  |  |  |  |  |  |  |  |
| N |  | 3950 | 3950 | 13769 | 13769 | 9810 | 9810 |  |  |  |  |  |
| *** p<0.01, ** p<0.05, * p<0.1  ECLS n rounded to the nearest 50 | | |  |  |  | | |  |  |  |  |  |

| **Appendix Table 2A: Maternal smoking during the pregnancy by maternal education** | | | | | |
| --- | --- | --- | --- | --- | --- |
|  |  | Maternal education | | |  |
|  |  | Low | Medium | High | p-value |
| *Proportion of mothers who smoked during the 3^rd^ trimester pregnancy* | | | | | |
| ECLS-B, US |  | 0.173 | 0.0849 | 0.0246 | <0.0001 |
| MCS, UK |  | 0.2957 | 0.1108 | 0.0344 | <0.0001 |
| Elfe, France |  | 0.2604 | 0.1278 | 0.0680 | <0.0001 |
|  |  |  |  |  |  |
| *Average number of daily cigarettes, conditional on any smoking (95% CI)* | | | | | |
| ECLS-B, US |  | 11.2 (9.9-12.4) | 8.9 (7.1-10.7) | 10.6 (3-18.3) |  |
| MCS, UK |  | 9.0 (8.7-9.3) | 6.6 (5.9-7.3) | 5.3 (4-6.5) |  |
| Elfe, France |  | 7.1 (6.8-7.4) | 4.6 (4.4-5.1) | 3.8 (3.6-4.06) |  |
|  | | |  |  |  |
